# A Longitudinal Prospective Study of Active Tuberculosis Among Patients in Tétouan, Morocco

**DOI:** 10.7759/cureus.81838

**Published:** 2025-04-07

**Authors:** Ayoub Ez-Zari, Laila Farouk, Cristina Vilaplana, Nadya Mezzoug, Khalid Bouti, Noureddine El Mtili

**Affiliations:** 1 Department of Biology, Laboratory of Biology and Health, Food Science and Health Research Team (UAE/U06FS) Faculty of Science, Abdelmalek Essaâdi University, Tétouan, MAR; 2 Department of Pulmonology, Unitat de Tuberculosi Experimental, Germans Trias i Pujol Research Institute and Hospital, Barcelona, ESP; 3 CIBER Respiratory Diseases (CIBERES), Instituto de Salud Carlos III, Madrid, ESP; 4 Department of Microbiology, Northern Metropolitan Clinical Laboratory, Hospital Universitari Germans Trias i Pujol, Badalona, ESP; 5 Department of Pulmonology, Mohammed VI University Hospital, Tangier, MAR; 6 Laboratory of Life and Health Sciences, Faculty of Medicine and Pharmacy of Tangier, Tangier, MAR

**Keywords:** 9-item patient health questionnaire (phq-9), anti-tb treatment, dots – directly observed treatment short-course, gad-7 score, prescription drug monitoring program, sputum culture conversion

## Abstract

Background: Tuberculosis (TB) continues to be a significant global health challenge, particularly in developing countries. Tétouan, Morocco, is among the most affected cities in the country; however, limited information is available on the impact of TB and its treatment on various aspects of patients' lives. This study aims to comprehensively analyze the clinical, microbiological, nutritional, and psychosocial characteristics of patients undergoing intensive pulmonary TB treatment in Tétouan.

Methods: We conducted a 1.5-year prospective study on patients with bacteriologically confirmed pulmonary TB during the intensive treatment phase. Clinical, social, psychological, and nutritional data were collected, and bacteriological monitoring was performed. Statistical analysis was conducted using SPSS software (IBM Corp., Armonk, NY), with a 95% significance level.

Results: Among the 125 surveyed patients (mean age: 37.5 years, male-to-female ratio: 3.8), 91 (73.2%) resided in urban areas, 19 (15.4%) had difficulties reaching a healthcare center, and 89 (71%) were newly diagnosed. Side effects were reported by 121 patients (97%), primarily muscle and joint pain. A balanced diet was maintained by 80 patients (63.9%) during treatment. Anxiety was observed in 102 patients (82%) and correlated with female gender, retreatment cases, smoking, side effects, and living in urban areas. Depression was experienced by 113 patients (91%), significantly associated with smoking, side effects, and retreatment cases. The sputum conversion rate was low (62.4%) and showed a significant correlation with symptom progression after two months of intensive treatment.

Conclusion: Consistent patient support throughout the entire treatment period is crucial to preventing dropout and treatment failure. Greater efforts are needed to strengthen economic, social, and psychological support for patients. Healthcare educational units play a key role in informing patients about nutrition, potential side effects, and nicotine withdrawal symptoms. These interventions are essential to enhancing adherence and improving overall treatment outcomes in Tétouan.

## Introduction

Tuberculosis (TB) remains a major global health concern. Due to its mainly airborne transmission, the extent of this pathology continues to be worrying worldwide. While TB incidence has declined by nearly 2% annually, the reduction in TB incidence from 2015 to 2020 was 11%, falling short of the 20% target set by the World Health Organization (WHO) [1]. In 2022, TB caused an estimated 1.3 million deaths worldwide, slightly lower than the 1.4 million deaths reported in 2021, bringing mortality rates closer to pre-pandemic levels. However, TB remains the second-deadliest infectious disease after COVID-19 and the 13th leading cause of death overall [2].

Recognizing these challenging statistics and the evolving nature of TB control, it becomes clear that a broader strategy is necessary. Efforts to control TB have historically centered on clinical treatment and monitoring, but this alone is insufficient for TB eradication. Growing recognition of the disease’s social, economic, and psychological impact has led to a shift toward a multidisciplinary approach, integrating nutritional, economic, and mental health support. This aligns with the United Nations’ Sustainable Development Goals (SDGs) and the WHO’s End TB Strategy (2016-2030), endorsed for global implementation, including in Morocco [3-5]. Additionally, TB treatment is associated with significant physical and psychological side effects, which can affect adherence, quality of life, and the risk of relapse, emphasizing the need to monitor treatment effectiveness and tolerability to prevent treatment failure and the emergence of drug resistance [6].

In Morocco, TB incidence has shifted from high to moderate levels, with a gradual decline from 102 per 100,000 inhabitants in 2015 to 93 per 100,000 in 2022. However, cases remain unevenly distributed, with the highest incidence in the northwestern region of Morocco, Tangier-Tétouan-Al Hoceima (111 per 100,000 inhabitants in 2022) [5,7]. Tétouan, part of this high-burden region, has shown a downward trend in TB cases over the past two decades, yet its incidence remains above the national average. This study aims to provide a comprehensive analysis of the clinical, microbiological, nutritional, and psychosocial characteristics of patients undergoing intensive pulmonary TB treatment in Tétouan, Morocco.

## Materials and methods

Study design and setting

This prospective, descriptive study was conducted over 18 months (January 2022 to June 2023) and included 125 patients diagnosed and treated for bacteriologically confirmed pulmonary TB. Patients were monitored at the Tuberculosis and Respiratory Diseases Diagnostic Center in Tétouan. Tétouan is located in the northwestern region of Morocco.

Ethical consideration

Data were anonymized and used exclusively for research purposes. The Institutional Review Board (IRB) of the Ethics Committee of the University Hospital of Tangier (CEHUT) reviewed and approved the research protocol (IRB No: AC112JV/2-025). The IRB approved the informed consent requirements, and written consent was obtained from all participants, as well as from parents or legal guardians for those under 18 years of age. To ensure privacy and confidentiality, all surveys were conducted in a separate room with only the surveyor and the participant present. Clear and concise instructions regarding the survey were provided both in the consent form and during the survey process. Additionally, participants were reassured that their responses, particularly on sensitive topics, would remain confidential to minimize social pressure and bias.

Participants’ inclusion criteria and sample size

Eligible participants were individuals with bacteriologically confirmed pulmonary TB who had completed the initial treatment phase, in accordance with WHO guidelines [8]. For minors (under 18 years) or individuals unable to respond independently, responses were obtained from a companion or legal guardian. The sample size was calculated using the Taro-Yamane formula, based on the total number of bacteriologically confirmed pulmonary TB cases recorded during the study period (N = 619), with an estimated margin of error of 0.08.

Data collection

Demographic data (age, gender, and residence), nutritional, and clinical data (initial symptoms, disease progression, and treatment-related side effects) were collected through patient interviews and complemented by medical records. Anxiety and depression were assessed using the Generalized Anxiety Disorder-7 (GAD-7) and Patient Health Questionnaire-9 (PHQ-9) scales, with a score exceeding 10 on either scale warranted clinical evaluation/intervention [9]. Additionally, patient-healthcare professional interactions were explored to gain insights into participants' overall well-being. Data collection occurred at baseline (diagnosis) and month 2 after treatment initiation.

Bacteriological status monitoring

Bacteriological status was determined at baseline (moment of diagnosis) using the GeneXpert MTB/RIF Ultra® (Cepheid India Private Limited, Gurgaon, India) test. At month 2, sputum samples were analyzed using two methods: Acid-Fast Bacilli (AFB) staining with the Auramine-O fluorescent technique and sputum culture on Löwenstein-Jensen medium, processed with Petroff’s method [10].

Statistical methods

Data were organized using Microsoft Excel, and statistical analyses were performed using SPSS 26.0 (IBM Corp., Armonk, NY, USA). Descriptive statistics (mean ± SD, median, and frequencies) were computed. Pearson’s correlation (r) and logistic regression models were applied to identify independent factors associated with dependent variables. Adjusted odds ratios (aORs) with 95% confidence intervals (CIs) were calculated, and statistical significance was determined with a p-value of ≤ 0.05.

## Results

Socioeconomic characteristics of patients, TB status, and comorbidities

The study included 125 patients, with a mean age of 37.5 ± 16.4 years and a sex ratio of 3.8, favoring men. The majority of participants (n = 92, 73.2%) resided in urban areas, while 94 (75%) lived in households with two to five individuals. Access to healthcare was reported as challenging by 20 patients (15.4%). A total of 81 (65.9%) participants were single, and 60 (48.4%) had completed secondary education. Additionally, 70 (55.8%) were employed, with 72 (57.7%) earning approximately €300 monthly.

Regarding smoking habits, 56 patients (44.4%) had never smoked, while 48 (37.8%) were former smokers, 26 (73%) of whom quit at the start of treatment. However, 22 (17.8%) continued smoking during the intensive treatment phase. Newly diagnosed TB cases comprised 89 patients (71%), and 101 (80.6%) had no comorbidities. The most frequently reported comorbid conditions were diabetes (n = 6, 4.8%) and anemia (n = 5, 4.0%) (Table 1).

**Table 1:** Demographic, social, clinical, nutritional, and treatment adherence characteristics of patients surveyed after completing the first two months of anti-tuberculosis treatment (N = 125) AFB**: Acid-fast bacilli smear using Auramine-O fluorescence staining; N: Total number of patients in the cohort; L-J: Loewenstein-Jensen medium culture; nⁱ: Number of patients included in the studied test.

Features	Number	(%)
Gender		
Men	99	79.2
Woman	26	20.8
Age groups (year)		
<15	2	1.6
15-34	66	53
35-54	32	26
>55	24	19
Matrimonial situation		
Single	81	65.9
Married	42	34.1
Habitat		
Urban	90	73.2
Rural	33	26.8
Smoking status during treatment		
Non-smoker	42	44.4
Smoker	17	17.8
Ex-smoker	36	37.8
Education level		
Primary	35	28.7
Secondary	59	48.4
University	16	13.1
Illiterate	10	9.0
Patient with stable employment	67	55.8
Revenue (monthly)		
Less than 200€	25	32.1
200€ - 400€	45	57.7
More than 400€	8	10.3
Number of individuals living with the patient		
None	11	9.2
2 to 5 people	90	75
More than 6	19	16
Patients with accommodation well-ventilated	105	85.4
Patients with difficulties reaching the health-care center for medication	19	15.4
Taking medicines before anti-TB treatment		
Antibiotics	63	51.2
Anti-inflammatory drugs	53	42.4
Anti-TB treatment compliance		
Dosage respected	119	96.7
Protocol respected	107	85.6
Daily treatment intake Respected	112	93.3
Patients nutrition’s habits During the 2-month treatment period		
Balanced diet	62	63.9
Changes in diet (for better convalescence)	28	28.9
Use of natural remedies in conjunction with TB treatment	30	30.9
Taking herbal remedies before anti-TB treatment (before TB diagnosis)	93	76.2
TB status		
New case	88	71
Relapse	30	24.2
Treatment failure	3	2.4
Lost to follow up	3	2.4
Comorbidities		
None	100	80.6
Diabetes	6	4.8
Anemia	5	4.0
Psychological disorders/Substance abuse	5	4.0
Arterial hypertension	2	1.6
Other	7	5.0
Clinical evolution after intensive treatment		
Fever Cessation	103	85
Weight gain (nⁱ = 115)	82	71.3
Sleep cycle regulation (nⁱ =103 )	82	79.6
No more night sweats (nⁱ = 117)	101	85.6
Improved general condition (nⁱ = 104)	49	47.1
Chest pain relief (nⁱ =86 )	70	81.4
Hemoptysis cessation (nⁱ = 42)	26	83.9
Shivering cessation (nⁱ = 37)	31	83.8
Sputum conversion		
AFB** smear negativity	78	62.4
Culture on L-J media negativity	76	60.8

Patients' nutritional status

Among all surveyed patients, 80 (63.9%) reported maintaining a balanced diet, while only 36 (28.9%) adjusted their dietary habits to optimize their recovery. Additionally, 39 (30.9%) stated that they used natural remedies alongside their treatment (Table 1).

Treatment before TB diagnosis and anti-TB treatment adherence

Before receiving a definitive TB diagnosis, 64 patients (51.2%) were prescribed antibiotics, while 53 (42.4%) received anti-inflammatory drugs. Following the initiation of anti-TB treatment, 121 patients (96.7%) adhered to the prescribed dosage, 107 (85.6%) strictly followed the therapeutic protocol, and 117 (93.3%) did not miss a single daily dose. Furthermore, 22 patients (17.6%) required hospitalization for at least one week at the start of treatment (Table 1).

A negative correlation was detected between smoking and treatment non-compliance using Pearson’s correlation coefficient (r): Failure to comply with prescribed dosage (N = 123, r = -0.192, p = 0.022); non-daily intake of treatment (N = 123, r = -0.199, p = 0.023).

Clinical and bacteriological monitoring

After two months of intensive treatment, AFB staining (Auramine O staining) remained positive in 47 patients (37.6%), while 49 (39.2%) had a positive culture result (Table 1). The initial diagnosis of TB was based on a range of symptoms, with generalized fatigue (n = 113, 90.4%) and weight loss (n = 113, 90.4%) being the most common. Other frequently reported symptoms included persistent cough (n = 105, 84%), night sweats (n = 99, 79.2%), fever (n = 89, 71.2%), and chest pain (n = 91, 72.8%). Hemoptysis was observed in 42 patients (33.6%), while dry cough was the least frequently reported symptom (n = 17, 13.6%).

Upon completing the intensive treatment phase, patients exhibited significant clinical improvement. Fever subsided in 75 patients (85%), while 80 (71.3%) experienced weight gain. Additionally, sleep disorders were resolved for 100 patients (80%), and 85 (85.6%) reported no longer suffering from excessive night sweats. Moreover, hemoptysis subsided in 35 out of 42 patients, representing 83.9%. All patients who initially experienced coughing reported a reduction in its intensity throughout treatment.

The patient’s clinical evolution was correlated with the sputum smear and culture conversion after two months of treatment; of which: weight gain, definitive cessation of night sweats, improvement in general condition without deterioration, and cessation of chest pain, were associated with a negative two-month check-up culture examination (Table 2).

**Table 2:** Logistic regression analysis of clinical symptom evolution after two months of treatment and delayed negativity of AFB staining and culture on L-J solid medium N**: Number of patients included in the statistical analysis; L-J: Loewenstein-Jensen culture medium; aOR: Adjusted odds ratio; 95% CI: 95% confidence interval; AFB staining: Acid-fast bacilli staining

Clinical evolution after 2 months of treatment	N**	Negative AFB smear staining test			Negative growth culture (L-J*) test		
		aOR	95% CI	P-value	aOR	95% CI	P-value
Cessation of fever	120	3.120	0.708 - 13.748	0.133	3.000	0.681 - 13.213	0.146
Weight gain	120	5.769	1.896 - 17.551	0.002	3.886	1.344 - 11.232	0.012
Sleep regulation	115	1.917	0.841 - 4.368	0.121	2.049	0.901 - 4.658	0.087
Cessation of nocturnal sweats	103	1.300	0.491 - 3.443	0.598	9.690	2.959 - 31.735	< 0.001
Improvement in overall clinical status (without deterioration)	118	2.944	1.028 - 8.425	0.038	7.008	2.118 - 23.185	0.001
Permanent cessation of chest pain	104	3.700	1.597 - 8.571	0.002	7.778	3.137 - 19.286	< 0.001
Hemoptysis cessation	36	1.375	0.210 - 9.015	0.740	5.500	0.839 - 36.059	0.076
Shivering cessation	86	2.385	0.700 - 8.125	0.165	1.556	0.488 - 4.960	0.455

Anti-TB treatment side effects

All patients experienced at least one treatment-related side effect. The most common were muscle pain (n = 78, 62.3%) and joint pain (n = 73, 58.2%). Skin manifestations were also frequent, including redness (n = 54, 43.4%), itching (n = 60, 48.4%), and rashes (n = 52, 41.8%). Additionally, some patients reported neurological effects, such as insomnia (n = 36, 28.7%), while one case of convulsions (0.8%) was recorded (Table 3). Several other side effects were reported, including loss of libido, headaches, dizziness, alopecia, and dehydration.

**Table 3:** Incidence of adverse side effects to first-line anti-tuberculosis drugs among surveyed patients (N=125) nF*: Number of female patients surveyed

Anti-TB treatment side effects	Number	%
Red colored urine	125	100
Skin reaction		
Redness	53	43.4
Itching	59	48.4
Eruptions	51	41.8
Muscle pain	76	62.3
Joint pain	71	58.2
Nausea	46	37.7
Abdominal pain	46	37.7
Vomiting	26	21.5
Diarrhea	19	15.7
Bloating	33	27
Anorexia	16	13.1
Jaundice	2	1.7
Edema	7	5.7
Purpura	1	0.8
Menstrual disturbance	4 (nF = 29)*	13.8
Extremities Hypoesthesia	0	0
Insomnia	35	28.7
Restlessness	4	3.3
Convulsion	1	0.8
Blurred vision	3	2.4

Psychological evaluation of patients

Nearly all patients (n = 119, 95.9%) expressed trust in their healthcare providers, and 91 (73.2%) described their relationship with them as positive. Instances of mistreatment were rare, with only three patients (2.4%) reporting having experienced it.

Before initiating treatment, 95 patients (76.1%) reported feeling emotionally unwell or moderately distressed. When asked about their reaction to being diagnosed with TB, 67 (53.7%) described the news as shocking, while 46 (36.6%) accepted it without concern. Interestingly, 15 patients (12%) felt reassured, as they had initially feared having a more severe illness than TB. 

The results indicated that 102 patients (82%) experienced some degree of anxiety, categorized as minimal (n = 21, 17%), mild (n = 65, 52%), moderate (n = 34, 27%), and severe (n = 4, 3%). Additionally, 114 patients (91%) suffered from at least mild depression, with severity levels ranging from mild (n = 60, 48%) to moderate (n = 41, 33%) and moderately severe (n = 13, 10%) (Table 4).

**Table 4:** Patients' anxiety and depressive states, along with healthcare professional–patient interactions (N = 125) GAD-7 scale *: 7-item Generalized Anxiety Disorder scale; PHQ-9 scale**: 9-item Patient Health Questionnaire depression scale

Categories	Number	%
Trusting the healthcare treatment team	118	95.9
Patient-healthcare provider relationship		
Good	90	73.2
Fair	30	24.4
Poor	3	2.4
Pre-treatment Psychological State		
Distressed	57	46.3
Moderately Distressed	37	30.1
No Emotional Concerns	18	14.4
No answer given	11	8.9
Emotional response upon diagnosis		
Accepted without Concern	45	36.6
Shocked / Surprised	66	53.7
Relieved (after fearing a more serious condition)	12	9.8
Anxiety level after intensive treatment using GAD7 scale*		
Minimal anxiety	21	17.0
Mild anxiety	64	52.0
Moderate anxiety	33	27.0
Severe anxiety	4	3.0
Depression level after intensive treatment using PHQ9 scale**		
Minimum depression	11	9.0
Mild depression	59	48.0
Moderate depression	40	33.0
Moderately severe depression	12	10.0
Severe depression	0	0

A total of 26 patients (20.8%) had an anxiety GAD-7 score of ≥11, and 39 patients (31.2%) had a depression PHQ-9 score of ≥11, both indicating eligibility for mandatory psychological intervention.

Logistic regression analysis showed that anxiety was significantly associated with female gender (p = 0.023), smoking during treatment (p = 0.002), and the occurrence of treatment-related side effects (e.g. nausea (p = 0.009), myalgia (p = 0.004) and arthralgia (p = 0.003)) and residing in urban areas (p = 0.040). Similarly, depression was significantly associated with the occurrence of side effects, smoking during treatment (p = 0.034), and TB retreatment status (p = 0.006) (Table 5).

**Table 5:** Association of demographic factors and treatment-related side effects with depression and anxiety during the intensive phase of tuberculosis treatment * Indicates a significant p-value; aOR: Adjusted odd ratio; 95% CI: 95% confidence interval; NS: Non-significant; PHQ-9: Depression Patient Health Questionnaire scale; GAD-7: Generalized Anxiety Disorder-7 scale.

Variables	Active depression (PHQ-9 >10)			Active anxiety (GAD-7>10)		
	aOR	95% CI	P value	aOR	95% CI	P value
Female gender	0.944	0.378 - 2.362	0.303	2.300	1.235 - 3.135	0.023*
Smoking during treatment	3.110	1.091 - 8.908	0.034*	5.437	1.848 - 15.998	0.002*
Retreatment cases	3.117	1.381 - 7.038	0.006*	1.914	0.787 - 4.654	0.152
Rural habitat	0.645	0.267 - 1.555	0.328	0.271	0.076 - 0.970	0.040*
Side effects appearance						
Eruptions	--	--	NS	2.452	1.023 - 5.877	0.040*
Nausea	2.505	1.159 - 5.416	0.020*	3.260	1.347 - 5.877	0.009*
Bloating	2.746	1.196 - 6.305	0.017*	--	--	NS
Vomiting	2.615	1.077 - 6.346	0.034*	--	--	NS
Abdominal pain	--	--	NS	2.667	1.112 - 6.392	0.028*
Myalgia	2.686	1.164 - 6.200	0.021*	6.462	1.820 - 22.945	0.004*
Arthralgia	2.909	1.286 - 6.580	0.010*	5.551	1.769 - 17.164	0.003*
Insomnia	--	--	NS	2.470	1.011 - 6.031	0.044*

## Discussion

The World Health Organization (WHO) has designed a multidisciplinary approach that prioritizes patient needs to deliver optimal care and follow-up [3]. It has been established that a satisfactory quality of life significantly enhances adherence to TB treatment [11].

This study is the first of its kind in Tétouan, analyzing a cohort of 125 TB patients. The average age was 37.5 years, with a male predominance and a sex ratio of 3.8. These findings align with the national findings [12]. While distance to healthcare centers was not a significant issue for most patients, given the presence of multiple centers within the urban area (where 73.2% (n = 90) of patients reside), 15.4% still face challenges related to this concern. Some patients required multiple modes of transportation to reach healthcare facilities for their medication, which is a risk factor highly linked to treatment non-adherence [13].

Employment status is a significant factor influencing treatment adherence and recovery. Among the patients, 44.2% (n = 55) were unemployed, and many of them reported job loss due to their physical inability to work, while others concealed their illness to avoid dismissal, which could further deteriorate their health and delay recovery. Financial instability remains a critical barrier in TB care, particularly in developing countries [14]. To mitigate this challenge, governmental and non-governmental organizations should implement financial support programs for TB patients, as recommended by the WHO [15]. In Tétouan, where pulmonary TB predominantly affects the working class, it is essential to adopt measures that reduce recovery time, given the economic importance of this population.

After two months of intensive treatment, the sputum conversion rate (62.4%) was lower than that in other countries such as India (82.4%) [16] and Tunisia (77%) [17]. Despite the low sensitivity of AFB sputum smear testing, it remains an essential tool for monitoring treatment response, assessing infectivity, and predicting relapse risk. Persistent AFB sputum smear positivity provides crucial insights into the evolution of bacillary load and the need for further investigation into adherence issues and treatment suitability [18]. In our study, several potential factors may explain the delayed sputum conversion. First, while patients reported satisfactory medication adherence, self-reported data may be subject to recall bias. Furthermore, Morocco previously implemented the directly observed therapy strategy (DOTS) from 1991 to 1996; however, this strategy was later partially replaced by the WHO's End-TB Strategy [19]. The shift away from DOT) has reduced daily interactions between patients and healthcare providers, which previously facilitated early intervention in cases of unexpected issues. Studies have shown that DOTS not only reduced smear positivity after four weeks but also improved treatment adherence and encouraged healthier patient lifestyles [20].

Early diagnosis is crucial for reducing mortality risk and can significantly enhance sputum smear conversion during treatment, as it allows for the timely initiation of effective therapy [21]. In this study, 76.2% (n = 93) of patients initially attempted self-medication before seeking professional care. Additionally, 51.2% (n = 63) received incorrect antibiotic prescriptions, and 42.4% (n = 53) were prescribed anti-inflammatory drugs, suggesting a potential lack of symptom-based TB diagnosis skills among healthcare professionals. These diagnostic delays may have contributed to the high positivity rates observed after intensive treatment [17].

Preventing TB transmission requires isolating bacilliferous patients either in hospitals or at home. A negative AFB smear test is a key factor for determining the end of quarantine [22]. In this study, 75% (n = 94) of patients cohabited with two to five individuals, emphasizing the need for patient education on preventive measures in households. Additionally, proper home ventilation is critical for minimizing airborne transmission [23], yet 14.6% (n = 20) of patients reported poor ventilation in their accommodations. A significant association was identified between symptom evolution during intensive treatment and sputum culture conversion. Indicators such as weight gain (p = 0.012), cessation of nocturnal sweats (p<0.001), and relief from chest pain (p<0.001) can help clinicians assess whether quarantine should be extended or ended. These findings align with those published by Wejse et al., who used clinical symptom progression to predict patients’ prognoses [24]. Cough persistence is a major indicator of contagiousness, with patients considered to be in the healing process only once the cough subsides. In our study, all patients reported a decrease in cough intensity, though it persisted. As a result, we were unable to assess the effect of cough evolution on sputum smear conversion.

Side effects from anti-TB treatment are common and vary in severity, potentially leading to treatment interruption or adjustment [25]. In this study, nearly all patients experienced at least one side effect, with myalgia and arthralgia being the most frequent. Treatment side effects were a primary reason for treatment non-compliance. Among the nine patients who admitted to failing to take their treatment daily, eight stated that the main cause was the appearance of side effects and their belief that their health was worsening because of the treatment. The negative impact of treatment is a major factor in treatment discontinuation and failure in Morocco [26].

Nutrition is crucial for TB recovery and the mitigation of side effects, as malnutrition worsens outcomes [27]. However, many patients lack nutritional awareness, highlighting the need for dietary support, particularly among those with comorbidities like diabetes and hypertension.

Smoking during TB treatment further complicates adherence. In this study, 17.8% (n = 17) of patients continued smoking during treatment, making them more susceptible to treatment non-compliance, which is consistent with previous findings [28]. Among former smokers (n = 36, 37.8%), 73% (n = 26) quit at the start of TB treatment. As a result, many struggled with withdrawal symptoms, adding to their TB symptoms and treatment side effects. Following WHO recommendations, professional support for smoking cessation is crucial to mitigate withdrawal-related distress among TB patients [29].

Psychological support should be provided to patients from the moment of diagnosis. In our study, 46.3% (n = 57) of patients reported experiencing frustration and emotional exhaustion at the time of diagnosis, primarily due to their physical inability to carry out daily activities.

Managing TB and its treatment can significantly influence patients' psychological well-being. Among the surveyed patients, 43% (n = 54) exhibited moderate to moderately severe depression, a prevalence consistent with findings from a meta-analysis (45.55%) [30]. Depression was associated with multiple factors, including TB status, commonly reported side effects, and smoking. Retreatment cases were three times more likely to develop depression (p = 0.006), aligning with a study conducted in Southwest Cameroon [31], possibly due to the psychological burden of undergoing a six-month or longer anti-TB treatment regimen again. Treatment-related side effects, particularly gastrointestinal and musculoskeletal manifestations, were significantly linked with depression, consistent with studies conducted in Ethiopia [32] and China [33]. Experiencing side effects daily can be mentally distressing and may contribute to the development of depressive symptoms [32,33].

Additionally, smoking was correlated with higher depression rates (p = 0.034), with smokers being three times more likely to experience clinical depression during intensive treatment. Many patients who continued smoking during treatment expressed a desire to quit but faced considerable challenges. This perceived inability to quit smoking may have further contributed to their emotional distress.

Anxiety was also prevalent, with 20.8% (n = 26) of patients requiring psychiatric intervention. This rate was lower than the 40% reported in Ethiopia [34] but higher than the 18.7% in China [33]. Women were more vulnerable to anxiety (p = 0.023), likely due to hormonal and social factors such as guilt, stigma, and discrimination [34,35]. Certain side effects, including skin rash (p = 0.040), muscle pain (p = 0.004), and digestive disorders (nausea, p = 0.009), were linked to increased anxiety. Numerous studies have corroborated these findings [32,34]. Additionally, smokers were five times more likely to experience anxiety attacks, as supported by a cross-sectional study conducted in South Ethiopia [34], underscoring the detrimental psychological impact of tobacco use during TB treatment. Interestingly, urban residents exhibited higher anxiety levels than rural residents (p = 0.040), suggesting that treatment sanctuaries outside metropolitan areas could enhance patient well-being.

This study had several limitations. First, during our study period, many patients missed their second-month follow-up appointment. In addition, the patients' medical records were not well documented; as a result, some data about comorbidities and the prescribed medications were missing, which led us to exclude several potential drug interactions from our analysis. However, this research provides a foundation for conducting more accurate and larger-scale studies.

## Conclusions

A comprehensive, well-integrated care policy is essential for improving TB treatment outcomes in Tétouan. This policy should adopt a multidisciplinary approach by incorporating psychologists, nutritionists, and social workers into TB care programs to address patients’ diverse needs. Additionally, patients who smoke should be closely monitored during nicotine withdrawal to prevent treatment non-adherent behaviors. Patients’ emotional support is crucial in the fight against TB and should begin as soon as a diagnosis is confirmed. The proven success of the DOTS strategy further underscores the need to strengthen the healthcare workforce, ensuring effective implementation without overburdening the current TB control system.
